# Supplementary material for: Epidemiology and Survival outcomes of HIV-associated cervical cancer in Nigeria
Source: medRxiv. 2023 Aug 13:2023.08.08.23293820. Preprint. [Version 1] doi: 10.1101/2023.08.08.23293820 (PMC10441483; doi:10.1101/2023.08.08.23293820)
Supplement: Supplement 1 [file NIHPP2023.08.08.23293820v1-supplement-1.pdf]

medRxiv preprint doi: https://doi.org/10.1101/2023.08.08.23293820; this version posted August 13, 2023. The copyright holder for this preprint
(which was not certified by peer review) is the author/funder, who has granted medRxiv a license to display the preprint in perpetuity.
It is made available under a CC-BY 4.0 International license .

Supplementary file: Scanned histopathologic images of cervical cancer with tumor grading

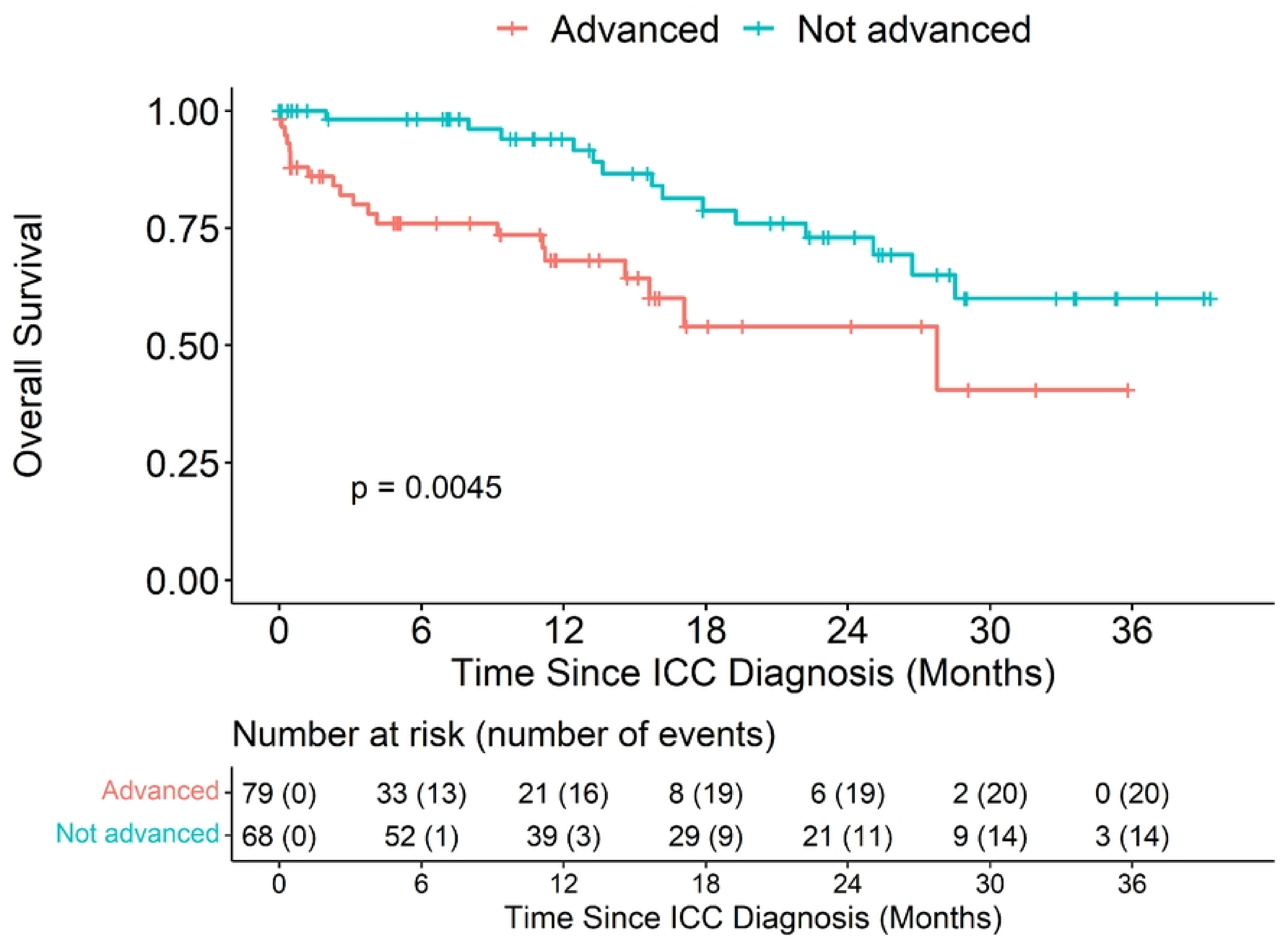
Advanced
Not advanced
Overall Survival
1.00
0.75
0.50
0.25
0.00
p = 0.0045
0
6
12
18
24
30
36
Time Since ICC Diagnosis (Months)
Number at risk (number of events)
Advanced
Not advanced
79 (0)
33 (13)
21 (16)
8 (19)
6 (19)
2 (20)
0 (20)
68 (0)
52 (1)
39 (3)
29 (9)
21 (11)
9 (14)
3 (14)
0
6
12
18
24
30
36
Time Since ICC Diagnosis (Months)

Figure
